# Potential health risks to disinfection workers from exposure to active substances in COVID-19 biocidal products

**DOI:** 10.1016/j.heliyon.2024.e28249

**Published:** 2024-03-20

**Authors:** Gihong Min, Jihun Shin, Dongjun Kim, Youngtae Choe, Jaemin Woo, Kil Yong Choi, Jangwoo Lee, Mansu Cho, Jongdae Lee, Jin-Sung Ra, Wonho Yang

**Affiliations:** a Department of Health and Safety, Daegu Catholic University, Gyeongbuk, South Korea; b Department of Environmental Energy Engineering, Anyang University, Anyang, South Korea; c Convergence Research Center for Big-data, Cheminet Ltd, Seoul, South Korea; d Department of Environmental Health Science, Soonchunhyang University, Chungnam, South Korea; e Eco-testing & Risk Assessment Center, Korea Institute of Industrial Technology, Ansan, South Korea

**Keywords:** COVID-19, Disinfection, Active substance, Hazard index, Disinfection worker

## Abstract

The importance of disinfection has recently been emphasized due to the increasing risk of the spread of infections such as coronavirus disease-2019 (COVID-19). In addition, disinfection for preventing the spread of COVID-19 is highly recommended. The increased use of biocidal products raises concerns regarding the potential health risks from exposure among disinfection workers. This study aimed to assess these exposure and health risks using questionnaires targeting disinfection workers who were exposed to the active substances in biocidal products used for disinfection during the COVID-19 pandemic. A follow-up survey was conducted among 271 disinfection workers for 10 working days within two weeks, and exposure factors with reference to disinfection were evaluated through interview-administered questionnaires. An exposure algorithm was used to evaluate the exposure of disinfection workers during disinfection. The hazard index (HI) was calculated by dividing the inhalation concentration obtained using the exposure algorithm and the dermal dose according to occupational exposure limits (OEL). A sensitivity analysis was conducted to identify the exposure factors with the greatest impact on the inhalation and dermal exposure algorithms. A logistic regression analysis was performed to verify the relationship with health effects and sociodemographic and exposure characteristics. The average number of disinfections performed during 10 working days was 17.5 ± 12.3 times. The type of disinfection work was divided into 2806 cases of COVID-19 prevention and disinfection and 1956 cases of regular pesticide application to prevent and remove any pests. The HI was ≥1, indicating a potential health risk, with the use of ethanol (6.50E+00), quaternary ammonium compounds (QACs; 1.49E+01), and benzalkonium chloride (BKC; 1.73E+00). Dermal exposure was more hazardous than inhalation exposure for 6 of the 11 active substances in biocidal products. The weight fraction and exposure time were the factors that most significantly influenced the inhalation and dermal exposure algorithms in the sensitivity analysis. Higher exposure concentrations were more likely to affect health (AOR: 3.239, 95% CI: 1.155–9.082). This study provides valuable information regarding the exposure and risk of disinfection workers to 11 biocidal active substances included in common disinfectants. Our results suggest that the use of ethanol, BKC, and QACs has potential health risks to disinfection workers, with a higher possibility of negative health impacts with increasing exposure concentration.

## Highlights

• Exposures of disinfectant workers to active substances in biocidal products have had potential health risks, although disinfection to prevent COVID-19 is essential.
• Disinfection workers could reduce the exposure by personal protective equipment, though the ethanol, QACs, and BKC represented potential health risks with an hazard index (HI) of ≥1.
• Health effects were significantly related to exposure concentration, while daily exposure time and occupation period were inversely related.

## 1 Introduction

Biocidal products are frequently used for infectious disease prevention in homes, workplaces, and public facilities [1]. Recently, the frequency of disinfectant usage to improve the safety of indoor environments safe has increased due to the COVID-19 pandemic [2]. Additionally, the U.S. Centers for Disease Control and Prevention (CDC) recommends disinfecting surfaces that humans come into contact with at least once a day to prevent the spread of COVID-19 disinfection [3].

Biocides can be used to control pests, such as flies, mosquitoes, and cockroaches that directly affect humans. It has been confirmed that biocides can act as a pesticide, disinfectant, or rodenticide depending on the disinfection target [4]. However, concerns are rising due to the harm caused by the misuse of and overexposure to biocides, as the indiscriminate use of biocidal products increased after the COVID-19 outbreak [5]. Chen et al. (2020) evaluated the acute effect of sodium hypochlorite use, demonstrating that the hypochlorite could react with internal and external ammonia to produce chloramine gas, which can cause symptoms, such as cough, shortness of breath, chest pain, wheezing, nausea, respiratory irritation, and pneumonia [6]. In addition, chronic health conditions such as asthma and rhinitis may arise from exposure to chemical by-products such as chloroform in improperly mixed products [7].

Disinfection workers may be directly exposed to the active substances in biocidal products when applied in indoor environments [8]. Therefore, professional disinfection workers would be exposed to high concentrations of active substances in disinfectant biocides and have more severe health risks compared to general public consumers or non-professional users [9]. Epidemiological studies have demonstrated that occupational exposure to disinfectant products was associated with an increased risk of asthma and rhinitis [10]. Sodium hypochlorite and quaternary ammonium compounds (QACs), as the most frequently used disinfectants, have been shown to adversely affect respiratory health [11,12]. Additionally, occupational exposure to pesticides and other biocides has been shown to increase the risk of developing thyroid cancer [13]. In general, the risks are related to local or systemic effects of the biocidal active substances through human inhalation and dermal exposure [14]. Exposure algorithms can be used to estimate the extent of exposure to active substances under biocide inhalation and direct contact via dermal exposure from direct contact [15]. Therefore, it is crucial to estimate the exposure and perform a risk assessment for disinfection workers using biocidal products.

Global regulations have been improved in recent years to protect human health and the environment from potentially harmful biocide products [16]. The European Union (EU) Biocide Product Regulation (BPR) and the Korea Household Chemicals and Biocide Safety Act (KBPR) were enacted to continuously improve and supplement the safe management of biocidal products [17,18]. The European Chemical Agency (ECHA) provides a list of the regulation related to biocidal active substances and biocidal product suppliers on its website (http://echa.europa.eu/regulations/biocidal-products-regulation/legislation) according to the EU BPR [19]. In addition, according to a voluntary agreement between the Ministry of Environment and chemical companies in Korea, the list of active substances in disinfectants was recently disclosed on the ecolife website (https://ecolife.me.go.kr) [20]. However, available information regarding the health risks of active substances in biocidal products commonly used by disinfection workers remains insufficient.

To fill this gap, this study aimed to develop a representative database of the exposure factors of disinfection workers to biocidal products and to conduct exposure and risk assessments based on the toxicity of the active substances contained in in these products. The selection of biocidal products was based on a survey of common products used on the job as reported by disinfection workers. The exposure and toxicity data obtained in this study can help to establish improved safety guidelines and facilitate further evaluation of the health effects of the active substances in biocidal products for disinfection workers.

## 2 Materials and methods

### 2.1 Interview questionnaire survey

An interview-based survey was conducted to determine which biocides were used by disinfection workers in metropolitan cities in Korea, including Seoul, Busan, Daejeon, Daegu, and Gwangju, between April and September 2021 during the COVID-19 pandemic. The population ratio of the five metropolitan cities was considered for recruitment, including and 271 disinfection workers from the Korea Pest Control Association. Follow-up interviews were conducted for the 271 disinfection workers for 10 working days within two weeks, and the number of disinfections per location was considered. The questionnaire sought information on exposure characteristics, such as the names of the product used, active substances, exposure time, usage frequency, room volume, type of disinfection sprayer, and daily usage amount. Additionally, the sociodemographic factors of disinfection workers and their health related symptoms, including eye, nose, throat, and skin irritation or pain during disinfection, were collected. A laser distance meter (SMART-300, Korea) was used to measure the room volume and the amount of biocidal product used was compared by measuring the mass of the product before and after its use balance. For the determination of other exposure factors, such as the settling velocity and airborne fraction, we followed the procedures recommended by the National Institute for Public Health and the Environment (RIVM) in the Netherlands [21].

This study was approved by the Institutional Review Board of Daegu Catholic University (IRB No. CUIRB-2022-056). In addition, all 271 participants signed a research consent prior to the survey.

### 2.2 Toxicity values of active substances in biocidal products

The test data for estimating the toxicity of active substances in the biocidal products used by disinfection workers were obtained from the research literature and related reports for humans and animals. The toxicological data used for estimating toxicity values to determine risk were reviewed, and the dose level that can cause adverse effects was determined by the point of departure (POD) values such as the no observed adverse effect level (NOAEL) and no observed adverse effect concentration (NOAEC). Toxicity values were applied to data obtained from inhalation, dermal contact, or oral ingestion. The toxicity values of chemicals in the biocidal products used by disinfection workers were determined with reference to ECHA, the U.S. Environmental Protection Agency (U.S. EPA) document, and the U.S. Registration Eligibility Decision (U.S. RED) documentation and related reports.

### 2.3 Assessing exposure and health risk

The main routes of exposure for disinfection workers are inhalation and dermal contact. Therefore, we applied inhalation and dermal exposure algorithms were applied with reference to the European Centre for Ecotoxicology and Toxicology of Chemicals and the RIVM report [21,22]. The spray algorithm was divided into continuous spraying and instantaneous release modes. Eq. (1) represents the model for continuous spraying using the ultra-low volume (ULV) spray machine, and Eq. (2) represents the model for instantaneous release using a high-pressure air pump manual sprayer. The airborne fraction (*F*_*air*_) was selected to be 0.3 and the droplet size was set to 28.1 ㎛ when a spray formulation was used [23]. The Stoke's settling velocity (*v*_*s*_) and room ventilation rate (*q*) were set to 154.02 m/h, and 0.6 h^−1^, respectively [23,24]. Eq. (3) considers the spraying duration when dermal exposure occurs through skin contact with active substances. The rate at which the product was applied to the skin (*R*_*der*_) for the constant rate algorithm was 269 mg/min for the ULV sprayer and 46 mg/min for the high-pressure air pump manual sprayer [25]. The loading time (*t*_*1*_) was set to be twice the value of the spraying time, considering the time taken for the substances to deposit [26]. The weight fraction (*W*_*f*_) and dilution factor (*D*) of the substance in the product were in the range of 0.013–83%, and 1–1000 times, respectively, as shown in Table 1.

(1) $$C_{a}=\frac{R_{s}\times W_{f}\times F_{air}}{V\times D\times \left(q+v_{s}/h\right)}\times \left(1-e^{-\left(q+v_{s}/h\right)\times t}\right)$$

where, *C*_*a*_ is the substance concentrations in the air indoors (mg/m^3^), *R*_*s*_ is the release rate of the sprayed product (mg/hr), *W*_*f*_ is the weight fraction of the active substance in the product (%), *D* is the dilution factor, *F*_*air*_ is the airborne fraction, *V* is the room volume (m^3^), *q* is the room ventilation rate (1/h), *v*_*s*_ is the Stoke's settling velocity (m/hr), *h* is the room height (m), and *t* is the exposure time (hr).

(2) $$C_{a}=\frac{A_{o}\times W_{f}}{V\times D}\times e^{-qt}$$

where, *C*_*a*_ is the substance concentrations in the air indoors (mg/m^3^), *Ao* is the product amount (mg), *Wf* is the weight fraction of the active substance in the product (%), *V* is the room volume (m^3^), *D* is the dilution factor, *q* is the room ventilation rate (1/h), and *t* is the exposure time (hr).

(3) $$L_{d}=R_{der}\times t_{1}\times \frac{W_{f}}{D}$$

where, *L*_*d*_ is the dermal load (mg), *R*_*der*_ is the rate at which the product is applied to the skin (mg/min), *t*_*1*_ is the loading time (min), *W*_*f*_ is the weight fraction of the active substance in the product (%), and *D* is the dilution factor.

**Table 1:** Biocidal products(disinfectants and pesticides) used by disinfection workers.

Biocidal product	Active substance	Weight fraction(%)	Dilution factor	
			Min	Max
Disinfectants	QACs	0.07–4.5	1	200
	Sodium hypochlorite	0.013–0.3	1	1
	BKC	9–20	200	300
	Oxone	49.7	100	100
	Ethanol	83	1	1
	Sodium dichloroisocyanurate	1.08	1000	1000
Pesticides	Zeta cypermethrin	5	150	150
	Deltamethrin	1.01–2.5	50	400
	λ-cyhalothrin	2.5	150	230
	Permethrin	30	100	600
	Etofenprox	10	200	200

The assigned protection factors (*APF*) were applied in inhalation exposure of disinfection workers, and the exposure concentration (*C*_exp_) calculated by the APF value of 10 was applied to the half-mask in Eq. (4) [27]. In addition, the dermal load (*L*_*d*_) was calculated by considering the absorption fraction and worker body weight in the dermal dose (*D*_*der*_), as represented by the exposure algorithm, which is expressed as Eq**.** (5) [28].

(4) $$C_{\exp}=C_{a}\times n\times \frac{t_{n}}{8}\times \frac{1}{APF}$$

where, *C*_exp_ is the exposure concentration (mg/m^3^), *C*_*a*_ is the substance concentrations in the air indoors (mg/m^3^), *n* is the frequency of use of biocidal products, *t*_*n*_ is the exposure duration per use (hr/use), and *APF* is the assigned protection factor.

(5) $$D_{der}=L_{d}\times abs\times N\times \frac{1}{BW}$$

where, *D*_*der*_ is the dermal dose (mg/kg/day), *L*_*d*_ is the dermal load (mg), *abs* is the absorption fraction (fraction), *N* is the frequency of biocidal product use (use/day), and *BW* is the body weight (kg).

The hazard quotient (*HQ*) of a non-carcinogenic active substance was derived by calculating *C*_exp_ and *D*_*der*_ using the exposure algorithm in Eq. (5) and dividing by the reference concentration (*RfC)* and reference dose (*RfD)*, respectively, as determined using Eq. (6) [29]. Finally, the hazard index (*HI*) was evaluated by the sum of inhalation hazard quotient (*HQ*_*inh*_) and the dermal hazard quotient (*HQ*_*der*_), expressed as Eq. (7). An HI of ≥1 indicated a potential health risk [29].

(6) $${HQ}_{inh}=\frac{C_{\exp}}{RfC},{\;HQ}_{der}=\frac{D_{der}}{RfD}$$

where, *HQ*_*inh*_ is the hazard quotient for inhalation (mg/m^3^), *C*_exp_ is the exposure concentration, *RfC* is the reference concentration (mg/m^3^), *HQ*_*der*_ is the hazard quotient for dermal exposure (mg/kg/day), *D*_*der*_ is the dermal dose (mg/kg/day), and *RfD* is the reference dose (mg/kg/day).

(7) HI=∑HQ

where, *HI* is the hazard index and *HQ* is the hazard quotient.

### 2.4 Statistical analysis

Statistical analyses were conducted using SPSS version 19.0 (IBM, Armonk, NY, USA), and differences with a p-value of <0.05 were considered to indicate a statistically significant difference. Chi-square tests and logistic regression analysis were performed to verify the relationship between health effects, such as eye, nose, throat, and skin irritation and sociodemographic and exposure characteristics. A sensitivity analysis was performed using @RISK ver. 8.2 software (Palisade Corporation, UK) to identify the exposure factors with the greatest impact on the inhalation and dermal exposure algorithm.

## 3 Results

### 3.1 Exposure factors for disinfection workers

Among the total 271 disinfection workers, there were 201 male workers (74.17%) and 70 female workers (25.83%). The exposure factors of the 271 disinfection workers (4762 total cases) were divided into disinfectant and pesticide procedures according to location. The mean and standard deviation values for the usage amount, exposure time, and room volume are presented in Table 2. The amount of disinfectant and pesticide usage was the highest in religious facilities (9120.00 ± 4032.86 g) and in public baths (5800.00 ± 5374.01 g), respectively. The exposure time for disinfectants and pesticides was the longest in religious facilities (37.77 ± 17.15 min) and in apartments (45.57 ± 36.49 min). The room volume for disinfection was the largest in gymnasiums (58,852.32 ± 105,116.98 m^3^) and was the smallest in detached houses (136.33 ± 92.37 m^3^). The average disinfectant usage amount (3934.91 ± 4603.15 g) was approximately 2.6 times higher than the average pesticide usage amount (1501.76 ± 2461.34 g). The average disinfectant and pesticide exposure times were similar at 16.64 ± 16.99 min and 15.69 ± 22.18 min. Compared to those for apartments and detached houses, the time of exposure to active substances in biocidal products was longer and the biocidal product usage amount was higher for public facilities.

**Table 2:** Exposure factors for biocidal products according to the workplace of disinfection workers.

Place		Biocidal product	Amount used (g/use)		Exposure time (min)		Volume (m^3^)	
			Mean	S.D.	Mean	S.D.	Mean	S.D.
Public facilities	General restaurants and pubs (N = 1420)	Disinfectant	1378.61	1983.09	9.27	7.72	438.81	2787.17
		Pesticide	1774.74	3246.84	12.63	14.65	386.78	1956.44
	Academy schools (N = 671)	Disinfectant	4870.37	4870.37	20.05	20.24	2982.69	11,607.25
		Pesticide	1586.43	1658.05	18.84	25.26	3123.82	8880.66
	Nursing homes (N = 451)	Disinfectant	3417.90	3037.57	13.93	11.20	2026.20	19,716.36
		Pesticide	1362.30	1454.21	18.86	14.45	1389.43	2114.23
	Gymnasiums (N = 131)	Disinfectant	5567.85	3666.85	22.07	13.80	11,301.94	15,684.71
		Pesticide	3450.00	3482.31	35.56	24.28	58,852.32	105,116.98
	Cafes and karaokes (N = 111)	Disinfectant	1308.93	1363.79	7.69	6.12	233.05	217.74
		Pesticide	997.69	1341.87	12.19	32.12	283.11	281.38
	Medical facilities (N = 67)	Disinfectant	3876.15	2950.89	16.64	11.99	2521.18	4513.73
		Pesticide	2730.67	1957.09	28.13	18.00	2327.99	3328.66
	Hypermarkets and markets (N = 60)	Disinfectant	4330.00	5827.68	17.25	14.30	3719.43	9537.55
		Pesticide	1874.55	1544.82	21.95	26.74	3457.69	10,481.39
	Religious facilities (N = 51)	Disinfectant	9120.00	4032.86	37.77	17.15	3695.93	6107.45
		Pesticide	4268.18	5513.09	26.36	155.68	584.20	547.77
	Viewing exhibition facilities (N = 38)	Disinfectant	4053.12	4064.73	20.32	15.90	5200.70	8803.02
		Pesticide	2166.67	1443.38	25.00	18.03	7044.85	7184.27
	Internet cafes (N = 26)	Disinfectant	4776.52	2437.80	20.20	9.76	916.10	416.48
		Pesticide	460.00	262.30	10.72	8.94	697.30	212.40
	Public baths (N = 14)	Disinfectant	3113.37	3092.27	13.74	12.59	946.45	1451.81
		Pesticide	5800.00	5374.01	27.50	17.68	743.80	350.63
Housing	Detached houses (N = 668)	Disinfectant	2531.46	2196.25	11.32	8.88	136.33	92.37
		Pesticide	609.77	1060.84	7.62	14.96	210.79	109.16
	Apartments (N = 186)	Disinfectant	1837.50	1739.41	9.00	5.42	223.39	178.97
		Pesticide	2701.97	2164.02	45.57	36.49	4026.36	3657.72
Offices (N = 666)		Disinfectant	5350.89	5513.94	18.88	17.89	2986.00	15,082.31
		Pesticide	1910.27	1683.23	23.00	21.09	3344.86	9297.21
Public transports (N = 202)		Disinfectant	6219.17	6797.08	27.07	27.39	917.42	2053.85
Total (N = 4762)		Disinfectant	3934.91	4603.15	16.64	16.99	2326.77	11,884.10
		Pesticide	1501.76	2461.34	15.69	22.18	1427.74	9529.97

S.D.: Standard Deviation.

### 3.2 Occupational exposure limits of active substances

Sodium hypochlorite and sodium dichloroisocyanurate emit chlorine gas, and NOAEC 1.5 mg/m^3^ was used as the toxicity value [30]. The toxicity values for benzalkonium chloride (BKC) and QACs were 0.22 mg/m^3^ and 0.1 mg/m^3^, respectively, according to the U.S. RED report and Korea Occupational Safety and Health Agency, respectively [31,32]. Oral toxicity values of 600 mg/kg/day and 2400 mg/kg/day were used for oxone and ethanol, respectively [33,34]. The toxicity values for deltamethrin, λ-cyhalothrin, permethrin, and etofenprox were determined according to the ECHA's pesticide assessment reports, and the toxicity value for cypermethrin was determined by referring to the U.S. RED [[35], [36], [37], [38], [39]]. Table 3 presents the *RfC* and *RfD* values applied for determining the occupational exposure limits (OELs) of disinfection workers based on the ECHA (2012) assessment factors (AF) [40].

**Table 3:** Occupational exposure limits according to dose-response assessments of active substances.

Active substance in biocidal product	Toxicity value	Occupational exposure limits		Reference
		Reference concentration (mg/m^3^)	Reference dose (mg/kg/day)	
Sodium hypochlorite	NOAEC = 1.5 mg/m^3^ (52 weeks/monkey, inhalation, sub-chronic)	1.51E-02	1.00E-01	[30]
		
Ethanol	NOAEL = 2400 mg/kg/day (90 days/rat, oral, sub-chronic)	8.64E-01	2.40E+01	[34]
		
Benzalkonium chloride	LOAEL = 0.22 mg/m^3^ (13 weeks/rat, inhalation, sub-chronic)	1.11E-03	2.00E-01	[32]
		
Oxone	LOAEL = 600 mg/kg/day (13 weeks/rat, oral, sub-chronic)	9.00E+00	6.00E+00	[33]
		
Quaternary ammonium compounds	LOAEL = 0.1 mg/m^3^ (4 weeks/rat, inhalation, sub-acute)	1.68E-04	6.67E-02	[31]
	
Sodium dichloroisocyanurate	NOAEC = 1.5 mg/m^3^ (52 weeks/monkey, inhalation, sub-chronic)	1.51E-02	3.08E+00	[30]
	
Deltamethrin	NOAEL = 1 mg/kg/day (52 weeks/rats, oral, sub-chronic)	4.29E-02	2.86E-02	[35]
		
Cypermethrin	NOAEL = 5 mg/kg/day (2 years/rat, oral, chronic)	1.50E-01	1.00E-01	[39]
		
λ-cyhalothrin	NOAEL = 0.5 mg/kg/day (52 weeks/dog, oral, sub-chronic)	2.14E-02	1.43E-02	[36]
		
Permethrin	NOAEL = 1000 mg/kg/day (90 days/rat, dermal, sub-chronic)	3.37E-03	1.00E+01	[37]
		
Etofenprox	NOAEL = 1000 mg/kg/day (4 weeks/rabbit, dermal, sub-acute)	2.01E-01	5.56E+00	[38]
		

### 3.3 Exposure assessment and sensitivity analysis

The *C*_exp_ and *D*_*der*_ values calculated using the exposure algorithm for 11 active substances in the biocidal products used by disinfection workers are presented in Table 4. The *C*_exp_ was the highest for ethanol (4.09E+01 mg/m^3^) and was the lowest for sodium dichloroisocyanurate (5.38E-05 mg/m^3^). Ethanol showed a high weight fraction of 83%, and its concentration was relatively higher than that of the other active substances when using the undiluted stock solution. The *D*_*der*_ was the highest for ethanol (4.24E+01 mg/kg/day) and was the lowest for λ-cyhalothrin (8.42E-04 mg/kg/day).

**Table 4:** Estimated inhalation (C_exp_) and dermal dose (D_der_) concentrations of the active substance of products for disinfection workers.

Active substance in biocidal product	C_exp_ (mg/m^3^)				D_der_ (mg/kg/day)			
	Mean ± S.D.	75th	95th	Max	Mean ± S.D.	75th	95th	Max
Sodium hypochlorite (N = 552)	3.07E-03 ± 1.08E-02	1.07E-03	1.26E-02	1.80E-01	9.77E-03 ± 3.32E-02	3.62E-02	4.20E-02	4.97E-01
Ethanol (N = 50)	4.09E+01 ± 5.32E+01	5.66E+01	1.14E+02	3.23E+02	4.24E+01 ± 7.90E+01	6.86E+01	2.17E+02	3.08E+02
Benzalkonium chloride (N = 119)	1.80E-02 ± 1.62E-02	2.02E-02	5.54E-02	5.80E-02	2.05E-02 ± 4.01E-02	9.60E-02	9.99E-02	1.27E-01
Oxone (N = 59)	3.33E-01 ± 1.08E+00	4.35E-02	2.28E+00	6.05E+00	1.29E-01 ± 3.04E-01	4.01E-02	1.06E+00	1.24E+00
Quaternary ammonium compounds (N = 2015)	2.44E-02 ± 1.19E-01	9.87E-03	1.47E-01	4.55E+00	2.67E-02 ± 7.08E-02	1.95E-02	1.52E-01	1.24E+00
Sodium dichloroisocyanurate (N = 11)	5.38E-05 ± 1.26E-04	2.20E-05	2.34E-04	4.32E-04	1.35E-03 ± 2.39E-03	1.42E-03	5.52E-03	7.85E-03
Deltamethrin (N = 658)	4.66E-02 ± 9.87E-02	3.06E-02	2.78E-01	1.14E+00	4.21E-03 ± 7.77E-03	5.48E-03	1.01E-02	8.97E-02
Cypermethrin (N = 838)	1.21E-02 ± 4.88E-02	8.80E-03	3.41E-02	9.06E-01	8.31E-03 ± 1.35E-02	8.14E-03	3.65E-02	1.14E-01
λ-cyhalothrin (N = 305)	6.90E-03 ± 1.53E-02	6.13E-03	3.15E-02	1.48E-01	8.42E-04 ± 2.25E-03	7.23E-04	2.38E-03	3.75E-02
Permethrin (N = 97)	3.34E-02 ± 3.70E-02	3.48E-02	9.80E-02	2.14E-01	4.72E-03 ± 2.57E-03	5.48E-03	1.01E-02	1.44E-02
Etofenprox (N = 58)	1.01E-01 ± 2.27E-01	7.17E-02	5.71E-01	1.32E+00	2.47E-02 ± 1.52E-02	3.07E-02	4.65E-02	1.08E-01

S.D.: Standard Deviation.

**Fig. 1:**
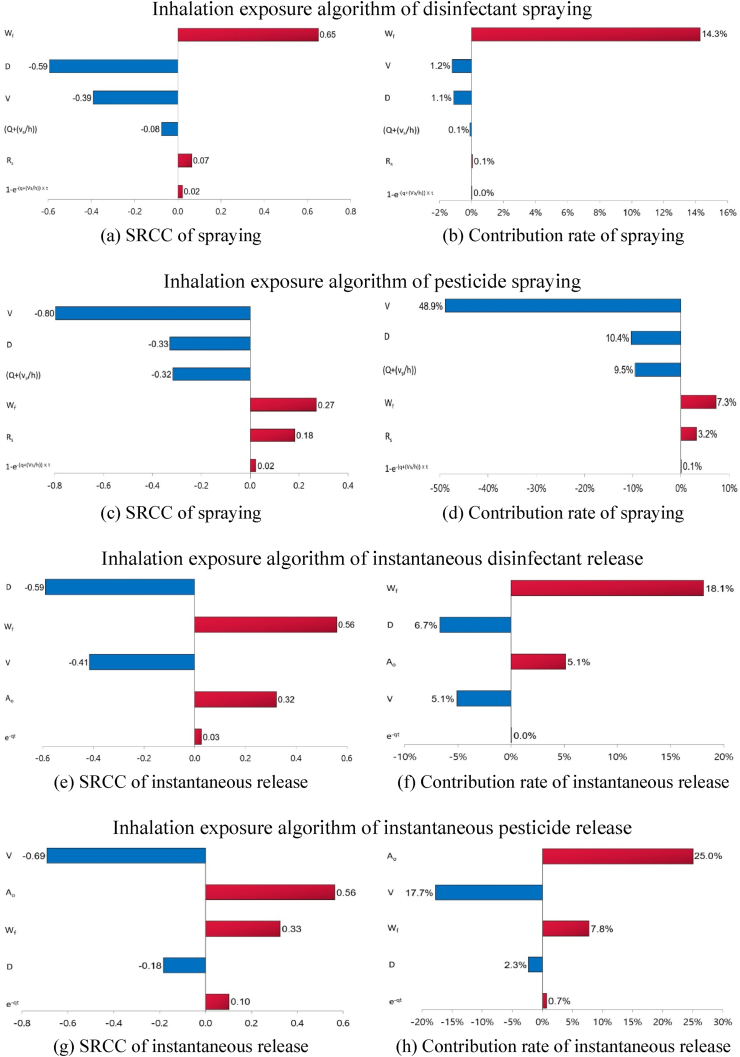
Spearman's rank correlation coefficients (SRCCs) and contribution rates for substance concentrations in the room air (C_a_) using sensitivity analysis.

**Fig. 2:**
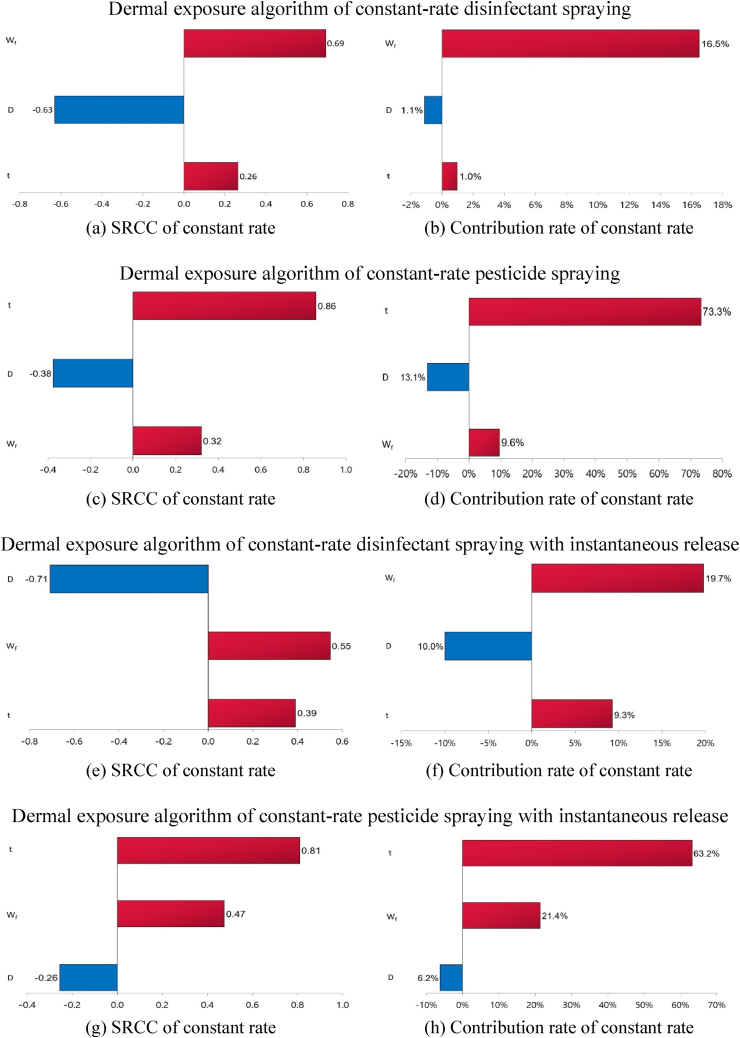
Spearman's rank correlation coefficients (SRCCs) and contribution rates for the dermal load (L_d_) using sensitivity analysis.

The Spearman's rank correlation coefficient (SRCC) of *C*_*a*_ was 0.65 for the *W*_*f*_ for disinfectant spraying and −0.80 for the *V* for pesticide spraying, with contribution rates of 14.3% and 48.9%, respectively (Fig. 1). As presented in Fig. 1, the disinfectants and pesticides applied via instantaneous release showed the greatest positive correlations in *A*_*o*_ and *W*_*f*_ for the SRCC of the *C*_*a*_ at 0.56 for both. The dermal exposure algorithm contribution rates were the highest for the *W*_*f*_ of disinfectants at 16.5% and 19.7% (Fig. 2). The dermal exposure algorithm contribution rates for pesticides were the highest for *t* at 73.3% and 63.2% (Fig. 2).

### 3.4 Health risk assessment

The *HQ*_*inh*_, *HQ*_*der*_, *HI*, and the *HQ*_*inh*_/*HQ*_*der*_ ratio for the 11 active substances are presented in Table 5. The *HQ*_*inh*_ was the highest for QACs (1.45E+01) and was the lowest for sodium dichloroisocyanurate (3.93E-04). The *HQ*_*der*_ was the highest for ethanol (1.80E+00) and was the lowest for sodium dichloroisocyanurate (4.64E-04), with a value < 1 indicating no potential health risks. Ethanol, QACs, and BKC represented potential health risks with an *HI* ≥ 1, and the remaining 9 active substances all had HI values < 1, ranging from 4.69E-04 to 9.92E-01. The *HQ*_*inh*_/*HQ*_*der*_ ratio was the highest for permethrin (2.55E+03) and was the lowest for sodium hypochlorite (1.85E-02). Additionally, the *HQ*_*inh*_/*HQ*_*der*_ ratio was <1 for 6 out of the 11 total active substances, indicating that dermal exposure had a more significant health effect.

**Table 5:** Estimated inhalation hazard quotient (HQ_inh_), dermal hazard quotient (HQ_der_), and hazard index (HI) of active substances in biocidal products used by disinfection workers.

Active substance in biocidal product	HQ_inh_				HQ_der_				HI (HQ_inh_ + HQ_der_)				HQ_inh_/HQ_der_ ratio
	Mean ± S.D.	75th	95th	Max	Mean ± S.D.	75th	95th	Max	Mean ± S.D.	75th	95th	Max	
Sodium hypochlorite (N = 552)	2.03E-02 ± 7.16E-02	7.08E-03	8.35E-02	1.19E+00	9.77E-02 ± 3.32E-01	3.62E-02	4.20E-01	4.97E+00	7.88E-03 ± 3.44E-01	4.11E-03	1.83E-02	1.98E-01	1.85E-02
Ethanol (N = 50)	4.76E+00 ± 6.04E+00	6.30E+00	1.30E+01	3.74E+01	1.80E+00 ± 3.23E+00	3.10E+00	8.89E+00	1.28E+01	6.50E+00 ± 5.71E+00	9.26E+00	1.36E+01	3.74E+01	6.49E-02
Benzalkonium chloride (N = 119)	1.62E+00 ± 1.45E+00	1.80E+00	4.99E+00	5.22E+00	1.03E-01 ± 1.99E-01	2.00E-01	5.00E-01	6.34E-01	1.73E+00 ± 1.60E+00	2.24E+00	5.47E+00	5.72E+00	1.56E+00
Oxone (N = 59)	3.84E-03 ± 1.19E-02	5.52E-04	2.33E-02	6.72E-02	2.19E-02 ± 5.00E-02	1.00E-02	1.76E-01	2.07E-01	2.56E-02 ± 5.18E-02	2.52E-02	1.77E-01	2.07E-01	1.56E-01
Quaternary ammonium compounds (N = 2015)	1.45E+01 ± 7.05E+01	5.88E+00	8.75E+01	2.71E+03	4.00E-01 ± 1.06E+00	2.92E-01	2.28E+00	1.86E+01	1.49E+01 ± 7.05E+01	6.26E+00	8.76E+01	2.71E+03	1.98E+02
Sodium dichloroisocyanurate (N = 11)	3.93E-04 ± 7.71E-04	2.38E-04	1.64E-03	2.86E-03	4.64E-04 ± 7.15E-04	6.90E-04	1.64E-03	2.55E-03	4.69E-04 ± 7.13E-04	6.90E-04	1.64E-03	2.55E-03	3.60E+00
Deltamethrin (N = 658)	1.09E-01 ± 2.30E-01	7.33E-02	6.48E-01	2.66E+00	1.47E-01 ± 2.71E-01	1.91E-01	3.53E-01	3.14E+00	2.56E-01 ± 3.63E-01	3.07E-01	9.51E-01	3.14E+00	8.27E-01
Cypermethrin (N = 838)	8.09E-03 ± 3.25E-02	5.88E-03	2.29E-02	6.04E-01	8.32E-02 ± 1.35E-01	8.32E-02	3.65E-01	1.14E+00	9.12E-02 ± 1.46E-01	9.02E-02	3.86E-01	1.16E+00	1.25E-01
λ-cyhalothrin (N = 305)	3.24E-02 ± 7.12E-02	2.96E-02	1.47E-01	6.92E-01	5.92E-02 ± 1.57E-01	5.55E-02	1.66E-01	2.62E+00	9.15E-02 ± 1.87E-01	8.43E-02	2.84E-01	2.69E+00	4.21E-01
Permethrin (N = 97)	9.92E-01 ± 1.09E+00	1.05E+00	2.84E+00	6.36E+00	4.70E-04 ± 2.55E-04	5.48E-04	9.97E-04	1.44E-03	9.92E-01 ± 1.09E+00	1.05E+00	2.84E+00	6.36E+00	2.55E+03
Etofenprox (N = 58)	5.15E-02 ± 1.11E-01	4.40E-02	2.74E-01	6.56E-01	4.41E-03 ± 2.70E-03	5.52E-03	8.30E-03	1.94E-02	5.58E-02 ± 1.11E-01	4.73E-02	2.79E-01	6.60E-01	1.59E+01

S.D.: Standard Deviation.

### 3.5 Factors influencing the health effects of biocidal products on disinfection workers

The health effects of disinfection workers who completed the questionnaires, such as eye, nose, throat, and skin irritation, were compared according to their sociodemographic and exposure characteristics. A health effect was considered to be present with at least one body irritation symptom reported. Among the tested predictors, the occupation period (p < 0.01) was the most significant factor in predicting the presence of health effects (Table 6). Table 7 shows the factors influencing the health effects of disinfection workers according to the logistic regression model. The adjusted odds ratio (AOR) was calculated to control for confounding variables such as sociodemographic and exposure characteristics. Health effects were not found to be significantly influenced by exposure concentration in Model I. Exposure concentration also was not a significant factor influencing health effects in Model II including sociodemographic characteristic variables. However, as the occupation period increased, the possibility of health effects decreased. In Model Ⅲ, all of the exposure characteristics that were significant in Table 6, including exposure concentration, occupation period, and daily hours of disinfection, were found to be independent predictors of exposure effects. The possibility of health effects increased when the exposure concentration was above 0.05 mg/m^3^ (AOR: 3.239, 95% CI: 1.155–9.082). The health effects decreased with an occupation period of 1.5–8 years of a career (AOR: 0.359, 95% CI: 0.141–0.913) and >8 years (AOR: 0.236, 95% CI: 0.106–0.526). In addition, the possibility of health effects decreased if the daily exposure time was >50 min (AOR: 0.428, CI: 0.193–0.951).

**Table 6:** Health effects according to sociodemographic and exposure characteristics (N = 271).

Variable	Category	Health effects (eye, nose, throat, and skin irritation)		χ^2^	p value
		Yes, n(%)	No, n(%)		
Sex	Male	62 (30.8%)	139 (69.2%)	0.565	0.452
	Female	25 (35.7%)	45 (64.3%)		
Age	20s	10 (24.4%)	31 (75.6%)	2.459	0.652
	30s	21 (35.6%)	38 (64.4%)		
	40s	33 (33.3%)	66 (66.7%)		
	50s	15 (36.6%)	26 (63.4%)		
	60s and over	8 (25.8%)	23 (74.2%)		
Smoking	Non-smoker	47 (32.6%)	97 (67.4%)	0.160	0.923
	Past smoker	16 (33.3%)	32 (66.7%)		
	Current smoker	24 (30.4%)	55 (69.6%)		
Underlying disease	Yes	3 (60.0%)	2 (40.0%)	1.819	0.177
	No	84 (31.6%)	182 (68.4%)		
Occupation period	<1.5 years	20 (30.3%)	46 (69.7%)	10.725	0.005**
	1.5–8 years	36 (25.5%)	105 (74.5%)		
	>8 years	31 (48.4%)	33 (51.6%)		
Type of disinfection sprayer	High-pressure air pump manual sprayer	28 (31.8%)	60 (68.2%)	0.459	0.795
	Ultra-low volume sprayer	24 (29.6%)	57 (70.4%)		
	Both	35 (34.3%)	67 (65.7%)		
Indoor air concentration	<0.10 mg/m^3^	21 (30.9%)	47 (69.1%)	0.187	0.911
	0.10–0.84 mg/m^3^	45 (33.3%)	90 (66.7%)		
	>0.84 mg/m^3^	21 (30.9%)	47 (69.1%)		
Daily hours of exposure time	<13 min	24 (34.8%)	45 (65.2%)	2.974	0.226
	13–50 min	37 (27.4%)	98 (72.6%)		
	>50 min	26 (38.8%)	41 (61.2%)		
Number of active substances used for disinfection	1	35 (31.3%)	77 (68.7%)	2.299	0.317
	2–3	41 (30.4%)	94 (69.6%)		
	4–7	11 (45.8%)	13 (54.2%)		
Personal protective equipment	Yes	64 (34.2%)	123 (65.8%)	1.245	0.264
	No	23 (27.4%)	61 (72.6%)		
Exposure concentration	<0.005 mg/m^3^	18 (26.9%)	49 (73.1%)	4.032	0.133
	0.005–0.05 mg/m^3^	52 (37.7%)	86 (62.3%)		
	>0.05 mg/m^3^	17 (25.8%)	49 (74.2%)		

N = 271 participants.

*p < 0.05; **p < 0.01.

**Table 7:** Adjusted odds ratios (AORs) and 95% confidence intervals (95% CIs) from logistic regression for the influence of sociodemographic and exposure characteristics on health effects.

Variable	Category	Model Ⅰ AOR (95% CI)	Model Ⅱ AOR (95% CI)	Model Ⅲ AOR (95% CI)
Exposure concentration	<0.005 mg/m^3^	(Ref)	(Ref)	(Ref)
	0.005–0.05 mg/m^3^	1.059 (0.489–2.292)	1.162 (0.519–2.602)	2.409 (0.538–10.786)
	>0.05 mg/m^3^	1.743 (0.910–3.339)	1.934 (0.955–3.917)	3.239 (1.155–9.082)*
Sex	Male		(Ref)	(Ref)
	Female		0.797 (0.364–1.743)	0.609 (0.247–1.501)
Age	20s		(Ref)	(Ref)
	30s		1.001 (0.327–3.061)	1.532 (0.424–5.538)
	40s		1.829 (0.661–5.058)	3.116 (0.953–10.184)
	50s		1.634 (0.622–4.293)	1.212 (0.410–3.577)
	60s and over		1.533 (0.496–4.738)	1.180 (0.347–4.011)
Smoking	Non-smoker		(Ref)	(Ref)
	Past smoker		0.890 (0.448–2.038)	0.775 (0.348–1.725)
	Current smoker		0.990 (0.455–2.625)	0.957 (0.352–2.598)
Underlying disease	Yes		(Ref)	(Ref)
	No		3.916 (0.595–25.783)	5.146 (0.749–35.375)
Occupation period	<1.5 years			(Ref)
	1.5–8 years			0.359 (0.141–0.913)*
	>8 years			0.236 (0.106–0.526)***
Type of disinfection sprayer	High-pressure air pump manual sprayer			(Ref)
	Ultra-low volume sprayer			0.965 (0.429–2.172)
	Both			1.007 (0.406–2.496)
Indoor air concentration	<0.10 mg/m^3^			(Ref)
	0.10–0.84 mg/m^3^			0.588 (0.142–2.438)
	>0.84 mg/m^3^			0.637 (0.251–1.617)
Daily hours of exposure time	<13 min			(Ref)
	13–50 min			0.801 (0.311–2.066)
	>50 min			0.428 (0.193–0.951)*
Number of active substances used for disinfection	1			(Ref)
	2–3			0.572 (0.187–1.750)
	4–7			0.585 (0.205–1.664)
Personal protective equipment	Yes			(Ref)
	No			0.748 (0.365–1.534)

N = 271 participants; AOR = adjusted odds ratio; CI = Confidence interval.

*p < 0.05; **p < 0.01; ***p < 0.0001.

## 4 Discussion

In this study, we determined the exposure factors of disinfection workers using a questionnaire and then risk of active substances in biocidal products was evaluated using an exposure algorithm. In addition, the health effects of disinfection workers according to the use of active substances in biocidal products were analyzed considering the sociodemographic and exposure characteristics included in the questionnaire,.

Intensified disinfection guidance during the COVID-19 pandemic requires increased disinfectant use in homes and public facilities [41]. In this study, disinfectant usage was approximately 2.6 times higher than pesticide usage. Therefore, it is probable that disinfectant usage for preventing the spread of COVID-19 and keeping indoor environments safe increased rapidly [42]. Many studies have indicated that the *RfC* values should be calculated using toxicological health effect assessment results, such as NOAEL and LOAEL, and that the results should be presented as OELs [[43], [44], [45]].

**Fig. 3:**
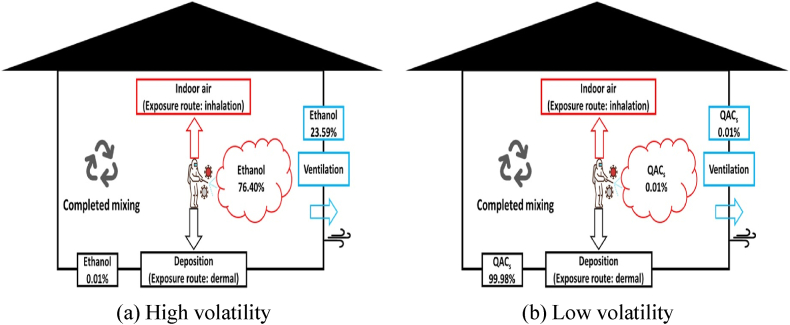
Mass balance of active substances in the modeled indoor environment.

When the biocidal active substances used in disinfection was sprayed, 99.99% of the ethanol with high volatility in the mass balance was volatilized, and 99.98% of the QACs with low volatility were deposited on the floor, as shown in Fig. 3. A previous study determined that 99.8% of highly volatile substances, such as *o*-phenylphenol, were volatilized, and the remaining 0.2% were precipitated [46]. Therefore, ethanol volatilizes according to its physicochemical properties, while QAC remains on the surface for a significant duration as a non-volatile compound [47].

The weight fraction had the greatest impact on the disinfectant spray exposure algorithm according to the sensitivity analysis. In general, disinfection with disinfectants more commonly involves the use of stock solutions compared to disinfection with pesticides; therefore changing the weight fraction and dilution factor can first be considered. Pesticide disinfection occurs over a large location in a single event, which would therefore be expected to have the largest contribution in terms of usage and room volume. The loading time of the dermal exposure algorithm showed a high contribution rate by assuming a value of twice the spraying time [25].

The calculated *HQ*_*inh*_ values for the 11 active substances used by disinfection workers indicated that biocidal products containing ethanol, QACs, and BKC had a potential health risk given that their *HQ*_*inh*_ values were all ≥1. This indicates that the disinfection method should be modified so that, the surfaces should instead be disinfected with a cloth soaked in disinfectant solution rather than by spraying the disinfectant to reduce the risk of exposure to biocidal products containing ethanol, QACs, and BKC [48,49]. The chemical components of indoor air rapidly change and the levels of volatile organic compounds (VOCs) increase when spraying ethanol with a weight fraction of ≥30%. Particle concentration produced health hazards during inhalation exposure, consistent with the results of this study [50]. Although the ethanol concentration was within the World Health Organization-recommended active substance concentration of 70–90%, the risk of ethanol exposure was nonetheless high because ethanol sprayed into the air presents a high exposure risk to the disinfection worker [51]. The extent of dermal absorption is determined by the physico-chemical properties of the active substance, with smaller molecules that exhibit good solubility in both water and fat generally penetrating the skin more effectively [52]. The HQ_*der*_ results of 11 active substances in the biocides used by disinfection workers were all <1, except for ethanol. The dermal exposure risk was low, as the skin was excluded for the exposure body surface area when wearing clothes or personal protective equipment (PPE) [28]. According to a study on the application, mixing, and loading exposure of pesticides, Thouvenin et al. (2017) showed that wearing of PPE provided up to 98.7% protection against dermal exposure [53].

The number of disinfection workers with health effects was approximately 5% higher in females than in males in our sample. Other studies have also determined that the incidence and health effects were higher in females than in males [54,55]. In the multi variable logistic regression analysis, Model Ⅲ showed that the risk of health effects increased with high concentrations and decreased as the exposure time and years of working increased. There are several possible explanations for these results. The inverse associations observed for the occupation period of the disinfection workers may be explained by a downward bias derived from the healthy worker survival effect [56,57]. Additionally, previous studies have demonstrated a limited ability to detect health effects due to low-level chronic exposure and periodic high-level exposure among humans who mix, load, and apply pesticides [58,59]. An additional probable explanation might be that a questionnaire, as an indirect exposure assessment method, may not accurately reflect the situation may not provide an objective response to health effects (Supplementary Material) [16]. Thus, information obtained from research subjects in a survey on health impacts might be inaccurate due to reporting bias, which can be predicted as an underestimation [60].

The significance of exposure and risk assessment for disinfection workers is increasing given the persistence of COVID-19. The main limitation of this study was that actual measurement values may differ from estimated values when using an algorithm that simulates the disinfection location. However, considering difficulty in obtaining actual measurement, presenting the health risk of each active substance in disinfection biocides using an exposure algorithm is crucial. Although we used room volume for the estimates in this study, methods that can obtain personal volumes such as 2 m^3^ and 5 m^3^ are necessary, because overestimation is possible in locations with large volumes [61].

## 5 Conclusions

Exposure and risk assessments for 11 biocidal active substances were conducted for disinfection workers during the COVID-19 pandemic using questionnaires. The average usage amount of disinfectants was approximately 2.6 times higher than that of pesticides and the exposure time was also longer for disinfectants. The *RfC* and *RfD* values applied to the OEL prediction for disinfection workers were derived. The exposure factors that most significantly impacted the inhalation and dermal exposure algorithms were weight fraction, usage amount, and exposure time. Based on our results, it is advisable to utilize a method by which the active substance is applied directly to surfaces rather than being sprayed into the air. Among the 11 active substances, ethanol, QACs, and BKC were determined to have the greatest potential health risks, as their *HI* values were above >1. Considering the logistic regression analysis result, a higher exposure concentration will increase the likelihood of health effects for disinfection workers, whereas a longer time in the occupation and longer daily disinfection hours can reduce the likelihood of health effects. Overall, the results of this study have implications for the development of guidelines on the use of safe active substances in biocidal products along with potential applications for strengthening public health safety measures.

## Funding statement

This study was supported by grants from the 10.13039/501100010700National Institute of Environmental Research (NIER-202100710001) and the 10.13039/501100003654Korea Environment Industry & Technology Institute through the Environmental Health Action Program funded by the Korea 10.13039/501100003562Ministry of Environment [Grant number 2021003320008].

## Data availability statement

Data will be made available on request.

## CRediT authorship contribution statement

**Gihong Min:** Data curation, Conceptualization. **Jihun Shin:** Writing – original draft, Supervision, Conceptualization. **Dongjun Kim:** Methodology, Investigation. **Youngtae Choe:** Resources, Methodology, Investigation. **Jaemin Woo:** Resources, Methodology, Investigation. **Kil Yong Choi:** Writing – review & editing, Writing – original draft, Conceptualization. **Jangwoo Lee:** Validation, Investigation, Data curation. **Mansu Cho:** Writing – review & editing, Methodology, Data curation. **Jongdae Lee:** Writing – original draft, Data curation. **Jin-Sung Ra:** Writing – original draft, Validation, Formal analysis. **Wonho Yang:** Writing – review & editing, Supervision, Resources, Funding acquisition, Conceptualization.

## Declaration of competing interest

The authors declare that they have no known competing financial interests or personal relationships that could have appeared to influence the work reported in this paper.
