# Supplementary material for: Potential health risks to disinfection workers from exposure to active substances in COVID-19 biocidal products
Source: Heliyon. 2024 Mar 20;10(7):e28249. doi: 10.1016/j.heliyon.2024.e28249 (PMC11002044; doi:10.1016/j.heliyon.2024.e28249)
Supplement: Multimedia component 1 [file mmc1.docx]

**Disinfection workers Exposure Survey**

| **Date** | (dd/mm/yy) | **Field**  **investigator** |  |
| --- | --- | --- | --- |
| **Name of respondent** |  | **Contract**  **information** | Telephone :  e-mail : |

**1. Information of disinfection worker**

| **Sex** | 1. Male 2. Female | **Smoking status** | 1. Non-smoker 2. Current smoker |
| --- | --- | --- | --- |
| **Age** | ( ) | **Smoking duration** | ( ) year(s) |
| **Body weight** | ( ) kg | **Average smoking per day** | ( ) cigarette(s)  ( ) a pack of cigarette |
| **Health effects**  **and others** | 1. Working period of disinfection?   ( ) year ( ) month   1. Have you ever felt the smell of biocide during disinfection?   ① Yes ② No  3. Have you had any symptoms of human irritation during disinfection?   1. ① Yes → ⑴ eye ⑵ nose ⑶ throat ⑷ skin 2. ② No   4. Do you know about the health risks of biocidal product?  ① Yes ② No  5. Do you have any underlying diseases?  ① Yes ② No | | |

**2. Place of the disinfection and working type**

| **Work type information and place** | | | |
| --- | --- | --- | --- |
| **Date** | (dd/mm/yy) | **Working time** | ( ) hr ( ) min |
| **Place** | 1. General restaurants and pub 2. Academy school 3. Nursing home 4. Gymnasium 5. Cafes and karaoke 6. Medical facilities 7. Hypermarket and markets 8. Religious facilities 9. Viewing exhibition facilities 10. Internet cafe 11. Public bath 12. Detached house 13. Apartment 14. Office 15. Public transport | Indoor or outdoor | 1. Indoor 2. Outdoor |
| **Place name** |  | | |
| **Place address** |  | | |
| **Place Area** | Width ( ) m / Length ( ) m / Height ( ) m | | |
| **Place volume** | ( ) m^3^ | | |
| **Working type** | 1. Disinfectant work 2. Pesticide work | **(Detail disinfection type)**   1. COVID-19 disinfection 2. General disinfection | |
| **Spraying time of ultra low volume (ULV) sprayer** | | ( ) hr ( ) min | |
| **Exposure time to the place** | | ( ) hr ( ) min | |
| **Spraying time of high-pressure air pump manual sprayer** | | ( ) hr ( ) min | |
| **Exposure time to the place** | | ( ) hr ( ) min | |

**3. Wearing personal protective equipment**

| **Personal protective equipment** | | | | |
| --- | --- | --- | --- | --- |
| **Protective**  **mask** | ☐ | ☐ | ▶ | 1. N 95, KF 94 2. Half mask 3. Powered air-purifying respirator (PAPR) 4. Others |
|  | Not  wearing | Wearing |  |  |
| **Protective**  **glasses** | ☐ | ☐ | ▶ | 1. Goggle safety glasses 2. Others |
|  | Not  wearing | Wearing |  |  |
| **Protective**  **Glove** | ☐ | ☐ | ▶ | 1. Rubber gloves 2. Others |
|  | Not  wearing | Wearing |  |  |
| **Protective**  **shoes** | ☐ | ☐ | ▶ | 1. Rubber shoes 2. Others |
|  | Not  wearing | Wearing |  |  |
| **Protective**  **cloth** | ☐ | ☐ | ▶ | 1. Disposable protective clothing 2. Others |
|  | Not  wearing | Wearing |  |  |
|  | 1. Short sleeved clothes ② Long sleeved clothes | | | |

**4. Information about biocide products**

| **Disinfectant work** | | | |
| --- | --- | --- | --- |
| **Product**  **name** |  | **Active substance**  **in biocidal product** |  |
| **Dilution**  **factor** | ( ) times | No dilution | |
|  | * Dilution place   1. On-site 2. Prepare in advance |  |  |
| **Usage**  **type** | 1. Spraying to air 2. Spraying to object 3. Wipe after spraying 4. Others | Spray type | |
|  |  | 1. Ultra low volume   (ULV) sprayer | 1. High-pressure air   pump manual sprayer |
| **Amount used** | | Before __________ mg | After __________ mg |
| **Exposure time** | | ___________ min | |
| **Pesticide work** | | | |
| **Product**  **name** |  | **Active substance**  **in biocidal product** |  |
| **Dilution**  **factor** | ( ) times | No dilution | |
|  | * Dilution place   1. On-site 2. Prepare in advance |  |  |
| **Usage**  **type** | 1. Spraying to air 2. Spraying to object 3. Wipe after spraying 4. Others | Spray type | |
|  |  | 1. Ultra low volume   (ULV) sprayer | 1. High-pressure air   pump manual sprayer |
| **Amount used** | | Before __________ mg | After __________ mg |
| **Exposure time** | | __ min | |

**5. Socio-demographic and working characteristics of disinfection workers using questionnaires**

| Variable | Category | N | % |
| --- | --- | --- | --- |
| Sex | Male | 201 | 74.17 |
|  | Female | 70 | 25.83 |
| Age | 20s | 41 | 15.13 |
|  | 30s | 59 | 21.77 |
|  | 40s | 99 | 36.53 |
|  | 50s | 41 | 15.13 |
|  | 60s over | 31 | 11.44 |
| Smoking | Non-smoker | 144 | 53.14 |
|  | Past smoker | 48 | 17.71 |
|  | Current smoker | 79 | 29.15 |
| Underlying disease | Yes | 5 | 1.85 |
|  | No | 266 | 98.15 |
| Occupation period | <1.5 years | 66 | 24.35 |
|  | 1.5–8 years | 141 | 52.03 |
|  | >8 years | 64 | 23.62 |
| Type of disinfection sprayer | High-pressure air pump manual sprayer | 88 | 32.47 |
|  | Ultra-Low Volume (ULV) sprayer | 81 | 29.89 |
|  | Both | 102 | 37.64 |
| Daily hours of exposure time | <13 min | 69 | 25.46 |
|  | 13–50 min | 135 | 49.82 |
|  | > 50 min | 67 | 24.72 |
| Number of active substance used for disinfection | 1 | 112 | 41.33 |
|  | 2–3 | 135 | 49.82 |
|  | 4–7 | 24 | 8.86 |
| Personal protective equipment | Yes | 187 | 69.00 |
|  | No | 84 | 31.00 |
